# Exploring New and Modified Rejection-Type Thresholds Using Cricket Snack Crackers

**DOI:** 10.3390/foods9101352

**Published:** 2020-09-24

**Authors:** Ryan Ardoin, Ross Romero, Brian Marx, Witoon Prinyawiwatkul

**Affiliations:** 1 School of Nutrition and Food Sciences, Louisiana State University, Agricultural Center, Baton Rouge, LA 70803, USA; rardoi7@lsu.edu (R.A.); rrome24@lsu.edu (R.R.); 2 Department of Experimental Statistics, Louisiana State University, Baton Rouge, LA 70803, USA; bmarx@lsu.edu

**Keywords:** sensory threshold, rejection threshold, no preference, cricket protein, probit regression

## Abstract

Recently, sensory threshold concepts have been expanded to include affective perceptions of foods and beverages, especially measures of product rejection; however, each threshold interpretation depends largely on the methodology employed. By substituting cricket powder for whole-wheat flour in snack crackers (0%, 5%, 10%, 15%, and 20%), we proposed modifications to two existing threshold methodologies: a modified consumer rejection threshold (M-CRT) using a 2-alternative choice (2-AC) preference test with “no preference” option; and a modified hedonic rejection threshold (M-HRT) based on less-than-neutral hedonic scores according to a one-sample *t*-test. We also proposed two new concepts: a rejection tolerance threshold (RTT) and an associated rejection range (RR), based on a binomial acceptability question and a probit regression model. These new methods provide more realistic interpretations of rejection-type thresholds based on user-defined allowable rejection levels, or “rejection tolerance” (for RTT), and a model-derived stimulus range (RR) to capture the rejection level of interest while providing flexibility for product formulation. All thresholds were investigated separately for color, flavor, texture and overall perception, elucidating differential sensorial effects of our novel ingredient on the food matrix. We suggest that conducting all demonstrated techniques in a single testing session gives a more expansive view of rejection thresholds while requiring minimal additional resources.

## 1. Introduction

Sensory thresholds represent the limits of auditory, visual, tactile, olfactory and/or gustatory capacities. Conceptually, a threshold is a value on a stimulus continuum (a boundary of sorts) where a perceptual change occurs [1]. This point is not fixed for all people at all times, so empirical threshold estimates are based on some mathematical models or probability distribution [2]. Conventional threshold measurements have modelled physiological responses to stimuli, but more recently, threshold methods have been devised for affective responses to food and beverages. We will refer to the later concepts, collectively, as rejection-type thresholds, as they originally involved tracking the deterioration of perceived product quality by increasing or decreasing the intensity of a specific stimulus [3,4]. While classical thresholds (absolute/detection, recognition, difference, and terminal thresholds) rely on perceived differences in attribute intensity [1,2], these differences may or may not trigger a change in affect for a given individual. It is implied, however, that for any perceptual effect of a stimulus to be observed, its concentration must surpass the detection threshold [3], and for subsequent changes in affect to occur, the just-noticeable-difference must be reached or exceeded [2].

Since Prescott et al. [3] introduced the first threshold of its kind, the consumer rejection threshold (CRT), using a paired-preference test, other authors have expanded the methodology to include measures of compromised acceptance (compromised acceptance threshold (CAT)) and sensory rejection of a food product (hedonic rejection threshold (HRT)) based on scores from the 9-point hedonic scale [4,5]. Most recently, Filho et al. demonstrated a similar approach based on increased liking (favored acceptance threshold, (FAT)) as well as analysis to effectively account for effects of two stimuli within a food matrix (hedonic thresholds methodology varying two stimuli (HTM_2s_)) [6]. Modifications to the CRT and HRT are presently proposed.

These affective sensory threshold methods have exhibited a wide range of valuable uses to the food industry, including but not limited to: informing limits on natural preservative use [7], determining practical sodium reduction levels [8], and mitigating negative effects of radiation on produce quality [9]. CRT measures have also been applied to compounds which may be desirable to some consumers at moderate levels, but eventually reach a breakpoint at higher concentrations [10,11], and even to stimuli for which specific lack of sensitivity is sometimes observed [11]. In these cases, our proposed modification, which accounts for “no preference” judgments, may help to characterize such market segments. Most importantly, since comparative assessments of preference do not always distinguish acceptable products from unacceptable ones, rejection-type threshold methodologies should include more explicit measures of rejection as the perceptual construct of interest [4].

Taking guidance from our previous study on appropriate product concepts for insect protein powder [12], the present vehicle for data collection was snack crackers formulated with increasing levels of cricket powder in place of whole-wheat flour, at up to 20% replacement. The resulting implications to insect-containing foods as a potential sustainable alternative protein source [13] are outside the scope of this paper. However, setting practical addition limits on a novel ingredient in food products adds to the growing list of real-world threshold applications. As the stimulus (cricket powder) effected changes in multiple sensory dimensions, separate thresholds were determined based on color, texture, flavor, and overall perceptions of snack crackers. Until recently [8], rejection-type thresholds had only focused on overall product quality.

The interpretation of each rejection-type threshold is contingent upon the affective dimension being measured, whether it be: preference (CRT) [3], relative degree of liking (CAT, FAT) [4,6], or absolute degree of liking (HRT) [4]. However, these projections of product rejection can be somewhat arbitrary, and as shown later, may not reliably align with more direct evaluations of acceptance. To more resolutely characterize rejection of a food product, we proposed use of a simple binary yes/no question. The resulting threshold estimates may offer more relevant guidance to food companies.

Additionally, we proposed two new rejection-type threshold concepts, the rejection tolerance threshold (RTT) and an associated rejection range (RR) using probit regression modelling. Along with demonstrating these new threshold methods using cricket powder in whole-wheat snack crackers, this research also presents modifications to two existing rejection-type threshold methodologies: a modified consumer rejection threshold (M-CRT) using a 2-alternative choice (2-AC) test [2] with “no preference” option; a modified hedonic rejection threshold (M-HRT) employing a one-sample *t*-test for added statistical rigor. These contributions should augment the expanding set of affective threshold methodologies available for sensory-driven product development.

## 2. Materials and Methods

This research involving human subjects was approved by the Louisiana State University (LSU; Baton Rouge, LA, USA) Agricultural Center Institutional Review Board (IRB# HE18-9 and IRB# HE 18-22).

### 2.1. Samples

The test samples used for this research were baked whole-wheat snack crackers modeled after a popular commercial product. Crackers were formulated with varying levels of cricket powder (*Acheta domesticus*, *Gryllodes sigillatus*; Griopro^®^, Midwest City, OK, USA) as a substitute for whole-wheat flour (*w*/*w*). The base formulation (whole-wheat flour, water, soybean oil, sugar, corn starch, salt, baking powder), without cricket powder, was used as a control. For threshold determination, test samples were formulated by substituting whole-wheat flour with cricket powder at levels of 0% (control), 5%, 10%, 15%, and 20%. Crackers were oven-baked at 350 °F (approximately 177 °C) and cooled at room temperature. Samples (two crackers each) were portioned into lidded plastic cups, labeled with three-digit blinding codes, and stored at room temperature overnight before testing.

### 2.2. Consumer Test

Consumers (*n* = 150) were recruited for participation from Louisiana State University campus (Baton Rouge, LA, USA). Sample presentation followed a balanced incomplete block design (B.I.B.; *t* = 4, *k* = 2, *r* = 3, *b* = 6, λ = 1) [14]. To allow for each cricket-containing sample (*t* = 4) to be directly compared to the control, each consumer was served two pairs of samples (*k* = 2 pairs), each consisting of one control and one stimulus sample. This resulted in six possible serving combinations (*b* = 6). Twenty-five replications of the design were carried out, such that 150 consumers participated (6 × 25 = 150 total panelists). Since each of the sample pairs occurred three times (*r* = 3) per replication, 75 observations per sample pair were obtained (3 × 25 = 75 observations per sample pair).

A balanced incomplete block (B.I.B.) design was selected in favor of a randomized complete block design to minimize sensory fatigue and adaptation [1]. Despite requiring more panelists to obtain the same number of observations, every consumer was only asked to try four total cracker samples (two pairs), instead of eight samples (4 pairs), from a randomly assigned serving presentation. Both the order in which each pair was evaluated and the order within each sample pairing (i.e., whether the control was evaluated first or second) were randomized in a counterbalanced fashion by the electronic questionnaire software (Qualtrics, Provo, UT, USA).

Testing was conducted in partitioned booths at the LSU Sensory Services Lab (Baton Rouge, LA, USA). Qualtrics online survey software (Qualtrics, Provo, UT, USA) was used for questionnaire presentation and response collection. Consumers were informed prior to testing that each sample may contain cricket. Although this knowledge could lead to expectation error [1] and lower thresholds based on negative attitudes toward entomophagy [12], it was deemed necessary to avoid any potential allergic reactions or unintended psychological distress. Samples were evaluated in terms of color, texture, flavor, and overall (liking/acceptability/preference), in that order. Consumers first rated each sample in the pair based on liking (a 9-point hedonic scale), followed by acceptability (a yes/no scale), for the above attributes. Once both samples in the pair were evaluated independently, consumers then reported preference (2-AC with “no preference” option) [2] between the two samples for each attribute. Unsalted crackers and water were served for palate cleansing.

### 2.3. Modified Consumer Rejection Threshold

The existing method for CRT [3] was modified from a forced-choice paired preference (2-AFC) to a 2-AC test with a “no preference” option [15]. The Thurstonian 2-AC modeling [16] was used to estimate the critical value for the modified consumer rejection threshold (M-CRT) as the lowest level of cricket powder substitution that would result in significant (α = 0.05) preference for the control. The hypotheses were as follows:

Ho: Proportion(preferring control) ≤ Proportion(preferring stimulus sample)(1)

Ha: Proportion(preferring control) > Proportion(preferring stimulus sample)(2)

A straight line was modeled (where x = % cricket powder and y = proportion of subjects preferring the control) between the first stimulus level to surpass the critical value and the preceding lower stimulus level. Since the critical value changed based on the number of “no preference” responses [14], another dashed line was constructed (where y = critical proportion of “prefer control” responses). The M-CRT was interpolated as the % cricket powder associated with the intersection of these two lines.

### 2.4. Hedonic Rejection Threshold and Modified Hedonic Rejection Threshold

The hedonic rejection threshold (HRT) was estimated following the established methods from Filho et al. [4], except using a B.I.B. serving protocol.

With the same data used to determine the HRT, the modified hedonic rejection threshold (M-HRT) was calculated based on results from a one-sample *t*-test to estimate the point at which mean liking scores (µ) for color, texture, flavor, and overall liking were, respectively, less than 5 (“neither like nor dislike” on a 9-point hedonic scale). The hypotheses were as follows:

Ho: µ ≥ 5(3)

Ha: µ < 5(4)

A simple linear regression [17] was used to model the calculated t-scores (*y*-axis, Equation (5)) as a function of cricket powder level (*x*-axis, as a substitute for whole-wheat flour).

*t*-value (calculated) = (x − 5)/(s/√75) (5)

The critical *t*-value of y = −1.67 (one tail, α = 0.05, df = 74) was superimposed to interpolate the M-HRT as the corresponding % cricket powder where mean liking would drop significantly below 5. This modification was expected to yield a more stringent criterion for assigning “rejection”, and hence, a more liberal threshold estimate, by employing a test which accounts for dispersion of the liking data.

### 2.5. Rejection Tolerance Threshold

To more directly evaluate sensory rejection, a simple yes/no forced choice question was asked (for color, texture, flavor, and overall acceptability), for example, “Is the flavor of sample 592 acceptable?” For this method, rejection of a given attribute was defined as a “no” response.

To determine the rejection tolerance threshold (RTT), a probit regression model [18] was fit using % cricket powder (x) as a predictor for the probability of rejection (y equaling a 0/1 “yes/no” response). As explained later, this approach to rejection-type threshold determination is based on a user-defined tolerance level (allowable proportion of rejection responses), and therefore dependent upon specific aims of the research, as opposed to a fixed critical value or hypothesis test. For the sake of this discussion, the RTT was demonstrated at a 25% rejection tolerance level. The RTT was thus estimated, from the model, as the % cricket powder expected to yield rejection from 25% of consumers.

### 2.6. Rejection Range

As the RTT is estimated from a generalized linear model, a confidence interval can be constructed around any point-estimate of the RTT [18]. The subsequent range of values bounded by this interval represents the rejection range (RR). To keep consistent with the α = 0.05 significance level used to demonstrate other thresholds tests, here we used a 95% confidence interval to estimate the RR.

All of the above-mentioned thresholds were measured separately in terms of color, texture, flavor, and overall perception of snack crackers. Data were analyzed using Microsoft Excel (2013), SAS (2013), and R software (2019) [19].

## 3. Results and Discussion

To facilitate the following discussion, Table 1 presents the growing list of affective threshold concepts and their abbreviations, including contributions of the current study.

### 3.1. Modified Consumer Rejection Thrshold

The CRT was the first affective threshold developed and originally used to find the level at which consumers would “reject” cork-tainted white wine [3]. The authors employed a forced-choice paired preference test (2-AFC) within a method of constant stimuli [2], comparing each tainted sample to a control. This particular concept has also been called simply “rejection threshold” (RjT) [21]. Despite its naming and intent, the CRT can only imply a comparative difference between two samples, without measures of magnitude or absolute acceptability, and therefore rejection should not be interpreted from this test alone.

The currently proposed modification, which separated M-CRT from existing CRT methodology, is the use of a “no preference” option, where, as opposed to a forced choice scenario (2-AFC) [1,2], M-CRT methods allowed judges to express a perceived tie or lack of preference between cricket-free and cricket-containing crackers. The M-CRT test would thus be consistent with a 2-alternative choice (2-AC) protocol [22]. For the M-CRT and subsequent threshold determinations, it was assumed that cricket powder had a directionally negative effect on affective perceptions of snack crackers, as did cork-taint in wine [3].

We know from existing literature that, when given the option, consumers do report ties, although their readiness to do so depends on (among other factors) the product category [23,24,25]. Properly accounting for ties [26] when they do exist may provide added resolution, help substantiate product superiority claims, and distinguish populations expressing lack of preference from those consisting of segments of consumers, each with a decided preference [15,22]. For attributes that exhibit optimum levels, even among niche consumer segments [10,11], methods such as Landscape Segmentation Analysis^®^ may provide added value [27]. Within the present range of cricket powder addition, the M-CRT methodology aimed to more clearly identify the shift from no overall preference or equal preference, to significant preference for the control crackers among consumers.

Instead of relying on a fixed critical value to assign the threshold as with the CRT, M-CRT used Thurstonian 2-AC modelling [16,28], which incorporates both a decision-making rule (cognitive parameter) and the degree of difference between samples (difference parameter) [22,29]. Treatment of “no preference” votes has included splitting them equally, splitting them proportionally, or dropping them all together [26]. Our analysis, on the other hand, incorporated them into the statistical modeling used to determine the M-CRT. As such, critical values for M-CRT hypothesis testing depended upon the proportion of ties [16], which ranged from 2.7% ties for overall preference (control vs. 20% cricket powder) to 25.3% ties for texture (control vs. 5% cricket powder). The M-CRT was interpolated for each respective attribute at the intersection of two straight lines, one connecting the observed percentage of consumers who preferred the control cracker, and the other connecting critical values for each level of comparison.

**Figure 1:**
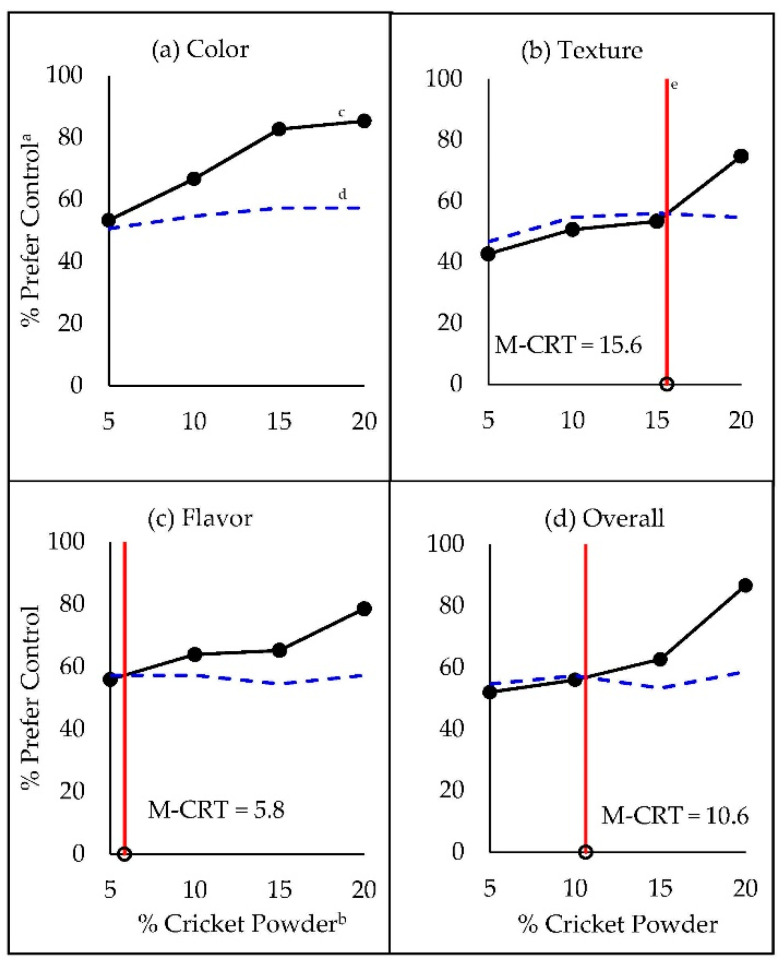
Modified consumer rejection thresholds (M-CRT) for cricket powder in whole-wheat snack crackers. Thresholds were measured based on 2-alternative choice (AC) paired preference tests of (**a**) color, (**b**) texture, (**c**) flavor, and (**d**) overall preference. ^a^ Percentage of consumers who reported preference for the control (0% cricket powder) sample. ^b^ Percentage of cricket powder used in place of whole-wheat flour (*w*/*w*) in cracker formulations. ^c^ Solid black lines connect points representing % of consumers preferring the control at each level of % cricket powder. ^d^ Blue dashed lines connect points representing Thurstonian 2-AC critical values (α = 0.05) at each level of comparison. ^e^ Vertical red lines represent the M-CRT (% cricket powder), estimated at the intersection of lines c and d (not found for color).

A precise estimate for the color threshold could not be determined, as it fell short of our lowest cricket powder substitution level of 5% (Figure 1a). Preliminary testing to verify that the minimum stimulus level performed similarly to the control in all aspects would have avoided this issue [29]. The M-CRT methodology did produce threshold estimates for flavor at 5.8% cricket powder (Figure 1c), followed by overall preference at 10.6% cricket powder (Figure 1d), and lastly for texture at 15.6% cricket powder (Figure 1b). An appropriate interpretation of this result would be, for example, that whole-wheat flour could be substituted at up to 10.6% with cricket powder before overall preference would be significantly affected, but the flavor imparted by cricket powder would be less preferred than that of whole-wheat beyond 5.8% substitution.

Not surprisingly, the affective dimension of preference proved to be the most sensitive, among those addressed, to the effects of cricket powder on snack cracker quality. However, these discontinuities in preference should not be confounded with conclusions of acceptance or rejection. Proceeding hedonic data will show that cricket-containing crackers were still liked at levels well exceeding their M-CRT estimates.

### 3.2. Hedonic Rejection Threshold and Modified Hedonic Rejection Threshold

The gap in interpretation between comparative ratings of inferior preference and independent measures of acceptance or rejection was noted by Filjo et al. when they proposed two additional affective thresholds, the CAT and HRT (originally called RT) [4]. Both were based on scores from the 9-point hedonic scale, and together, along with the more recently defined FAT and HTM_2s_ [6], were appropriately termed hedonic threshold methodology (HTM) [20]. To this list, we add a modified approach to the HRT, calling it simply a modified hedonic rejection threshold (M-HRT). As opposed to our M-CRT, which suggests an alternative approach to the existing methodology, the HRT and M-HRT offer slightly different interpretations and can thus be obtained jointly from the same data set.

Although the words “liking” and “acceptance” are often used interchangeably, the present discussion describes data from a labeled 9-point hedonic scale (anchored at 1 = dislike extremely and 9 = like extremely) in terms of liking, and later, responses to a binomial yes/no questions about product acceptability in terms of acceptance (for “yes” responses) or rejection (for “no” responses). Using the 9-point hedonic scale, Filho et al. [4] designated scores below the neutral category of 5 (neither like nor dislike) as “where rejection begins to occur”. In essence, the HRT methodology uses a simple linear regression (SLR) [17] to estimate the stimulus level (x) corresponding to a hedonic score of 5 (y). The resulting threshold does not, however, account for any spread around the point-estimate. The M-HRT, on the other hand, imposes a one-sample *t*-test to evaluate a one-sided hypothesis (Ho: µ ≥ 5, Ha: µ < 5). The associated question of interest is then related to the point where “liking drops significantly below 5”. Following HTM protocol, which asked respondents to evaluate their liking of each product monadically, we estimated the HRT and M-HRT for cricket powder in snack crackers.

**Figure 2:**
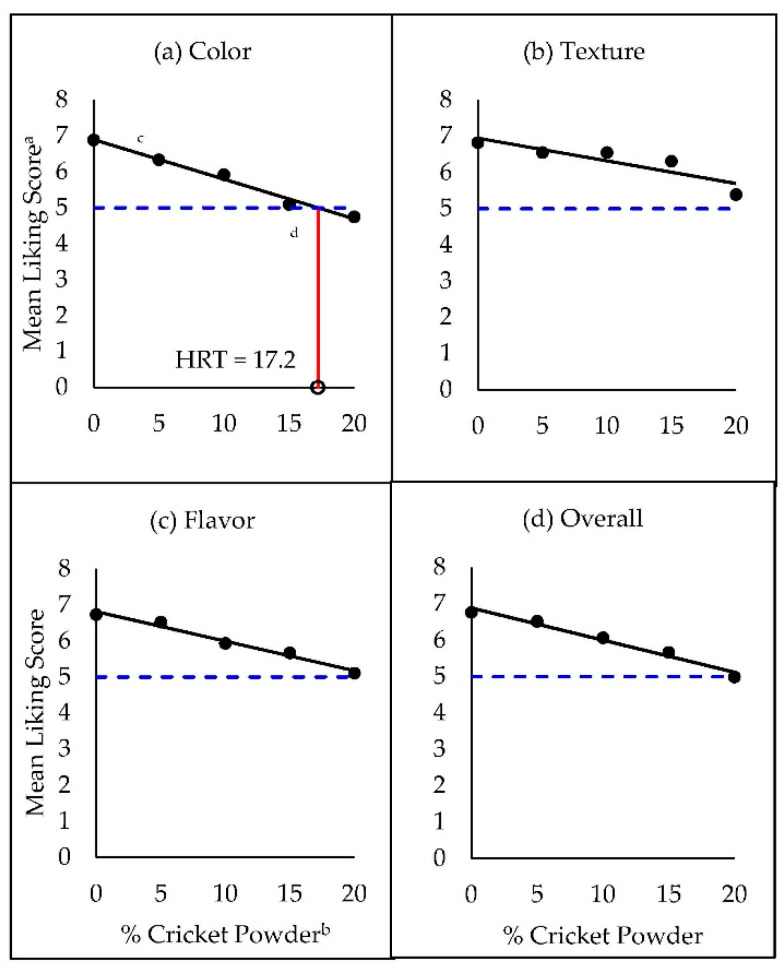
Hedonic rejection thresholds (HRT) for cricket powder in whole-wheat snack crackers. Thresholds were measured based on liking (a 9-point hedonic scale) of (**a**) color, (**b**) texture, (**c**) flavor, and (**d**) overall liking. ^a^ Mean liking scores from a 9-point hedonic scale. ^b^ Percentage of cricket powder used in place of whole-wheat flour (*w*/*w*) in cracker formulations. ^c^ Black solid line represents a simple linear regression of liking scores (y) as a function of % cricket powder (x), and black dots represent observed mean liking scores for each treatment. ^d^ Vertical red line represents the HRT, which was interpolated as the % cricket powder associated with a predicted hedonic score of 5 (blue horizontal dashed line) from the simple linear regression (not found for texture, flavor, or overall liking).

Whereas preference-based M-CRT estimates ranged from <5% to 15.6% cricket powder depending on the attribute evaluated (Figure 1), the mean liking scores for all attributes remained favorable (above 5) approaching 20% cricket powder. In fact, the only attribute for which the HRT could be determined within our stimulus range was color (HRT_color_ = 17.2% cricket powder; Figure 2a). Based on the HRT, color-liking of snack crackers would not be expected to drop below the neutral category until >17.2% whole-wheat flour substitution with cricket powder. In the present case, exceeding 20% wheat flour substitution without the addition of other functional ingredients would be challenging due to the lack of dough cohesion when forming the thin snack crackers.

**Figure 3:**
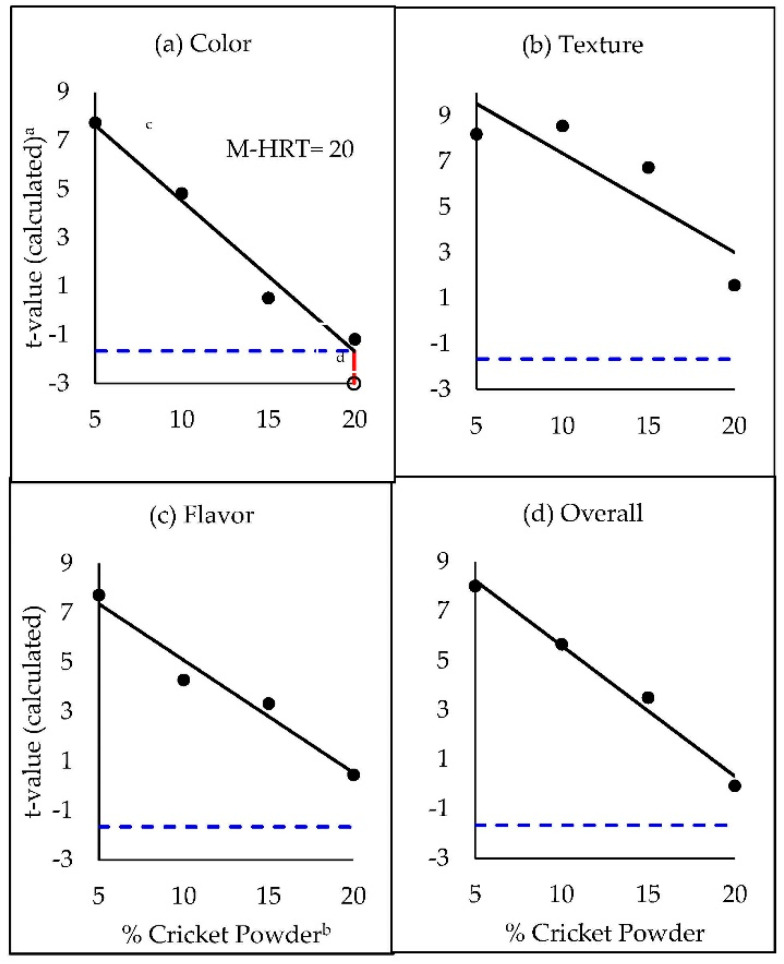
Modified hedonic rejection thresholds (M-HRT) for cricket powder in whole-wheat snack crackers. Thresholds were measured based on liking (a 9-point hedonic scale) of (**a**) color, (**b**) texture, (**c**) flavor, and (**d**) overall liking. ^a^ T-values were calculated from difference of mean liking scores (a 9-point scale) from a score of 5. ^b^ Percentage of cricket powder used in place of whole-wheat flour (*w*/*w*) in cracker formulations. ^c^ Black solid lines represent a simple linear regression of *t*-values (y) as a function of % cricket powder (x), and black dots represent observed *t*-values at each treatment level. ^d^ Vertical red line represents the M-HRT, which was interpolated as the % cricket powder associated with a critical predicted *t*-value (α = 0.05) of 1.67 (blue dashed horizontal line) from the simple linear regression (not found for texture, flavor, or overall liking).

To determine M-HRT levels, a new SLR was fit to model calculated t-scores (y, based on the transformed difference of each mean from 5) as a function of % cricket powder (x) in snack crackers. The new M-HRT was interpolated at the intersection of the regression line with the critical *t*-value of −1.67, (74 df, one sided α = 0.05). Again, color was the only attribute for which the hedonic threshold was obtained (M-HRT_color_ = 19.97% cricket powder, Figure 3a). As expected from this more strenuous criterion, the M-HRT methodology yielded a more liberal (higher) threshold estimate than the existing HRT protocol, at approximately 20% cricket powder.

Intuitively, the extra statistical rigor from a significant *t*-test may deliver added confidence that the M-HRT dependably predicts stimulus levels (based on hedonic scores) consistent with rejection. By plugging the calculated M-HRT_color_ value of 19.97% cricket powder into the SLR equation (Equation (6), R^2^ = 0.98) used to determine HRT_color_:

(6) $$\hat{y}=\;-0.6214x+10.745,$$

color liking is estimated to be 4.7. Analyzing the raw liking data, we find that the mean liking score for color at 20% cricket powder substitution was in fact 4.7. While 4.7 is not a valid response on the categorical 9-point hedonic scale (one must choose either 4 or 5), Gamba et al. [30] evaluated the performance of continuous unstructured and hybrid line scales with HTM and suggested the hybrid scale as an alternative to the 9-point hedonic scale, in which case, our proposed modification could still be applied. Nevertheless, binomial acceptance data suggested that the criteria consumers used for where acceptance ended and rejection began on the 9-point scale differed among individuals, and from the previously assigned cutoff of 5. The following discussion will turn to modeling rejection in a less arbitrary fashion.

### 3.3. Rejection Tolerenace Threshold and Rejection Range

For each sample, consumers were also asked to report (“yes” or “no”) whether the sample was acceptable overall and in terms of color, texture, and flavor. By compiling all these responses (2400 total) and pairing them with their associated hedonic scores, it became evident that consumers held different standards. If we consider a “no” acceptability response as self-reported rejection, then 3.2% of 6 scores and 6.3% of 5 scores were associated with rejection, and at the hedonic category of 4, the observed rejection rate was 45%. This implies that, even with a “dislike slightly” score, samples were still deemed acceptable 55% of the time (data not shown).

Admittedly, consumers’ perceptual responses to food and beverages are neither uniform nor consistent [31]. This problem has led to doubt about the validity and usefulness of empirical thresholds [1,2] and whether or not they actually exist in practice [32]. To overcome this ambiguity of hedonic threshold estimates, the currently proposed rejection tolerance threshold (RTT) and rejection range (RR) relied on the binomial yes/no responses for acceptance, where rejection of an attribute was defined as a “no” response. Given that binary responses often follow an “S”-shaped curve, linear regression models may not be appropriate [17]. Modeling thus requires a nonlinear link function, where in the present case, the slope of the effect depended on the amount of cricket powder present in snack crackers. The new methodologies aimed to provide more realistic interpretations of sensory-derived rejection limits by describing the tendency of consumers, within a given population, to accept or reject products as following a cumulative normal-type tolerance function. A similar approach is often used in medical and toxicological research to explore effective levels of a drug or lethality of poisonous substances [18].

To estimate the RTT, probit regression was used to model the probability of rejection as a function of increasing cricket powder. Fechner first applied a cumulative distribution curve to explain variability in perceptual responsiveness [33]. The current nuanced application of the probit model to affective thresholds allows investigators to begin with an allowable proportion of rejection responses, or a “rejection tolerance”, and estimate the stimulus level associated with such an outcome [18]. The resulting RTT is thus based on a user-defined tolerance of practical significance, rather than a critical statistical value or arbitrary boundary. Additionally, the estimate is derived considering the full range of data.

In one classical approach to thresholds [1,2], the point at which crackers would be rejected 50% of the time (i.e., RTT_50_) could be estimated by the mean of the distribution [18]. Other options would be to assign the threshold at the response probability 50% over that predicted by chance (here, 75% rejection) or based on the significant proportion of responses from a binomial test (here, corresponding to 60% rejection) [2]. However, these levels of rejection were neither achieved within the present study nor do they seem practical. An advantage of the proposed protocol is that the RTT can rather follow some investigator-defined tolerance, based on consumer self-reporting and according to the researcher’s objectives or company’s goals. Conversely, acceptance could be modeled as the proportion of “yes” responses, equivalent to 1 minus the probability of rejection.

**Figure 4:**
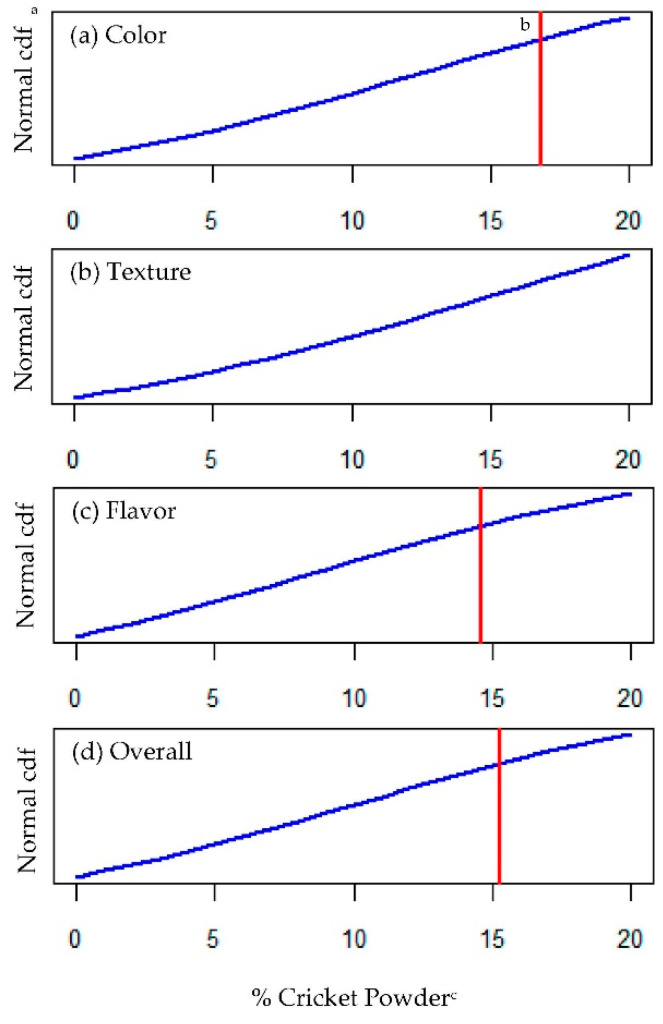
Rejection tolerance threshold_25%_ (RTT_25_) for cricket powder in whole-wheat snack crackers. Thresholds were measured for (**a**) color, (**b**) texture, (**c**) flavor and (**d**) overall acceptance using probit regression, based on 25% rejection tolerance, where the cumulative left-tail probability = 0.25 (represented by an area under blue line). ^a^ Normal cumulative distribution functions (cdf) were based on probit regression models, where the left-tail area under blue line represents the estimated probability of rejection (acceptability = “no”) as a function of % cricket powder. ^b^ Red vertical line represents the RTT_25_ as the predicted % cricket powder associated with 25% rejection (acceptability = “no”) from probit models (not found for texture). ^c^ Percentage of cricket powder used in place of whole-wheat flour (*w*/*w*) in cracker formulations.

For illustrative purposes, let us suppose that a food company is willing to accept 25% rejection of their cricket-containing snack crackers. Based on this hypothetical rejection tolerance of 25%, the RRT_25_ was determined for flavor at 14.6%, overall acceptability at 15.3%, and color at 16.8% whole-wheat flour substitution with cricket powder (Figure 4a,c,d, respectively). Consistent with the other affective measures, texture quality held up best to cricket powder addition, and no RTT was found for texture below 20% substitution (Figure 4b). From our current consumer sample, we found that the product would begin to reach the given rejection tolerance (25%) at around 15% whole-wheat flour substitution with cricket powder.

**Figure 5:**
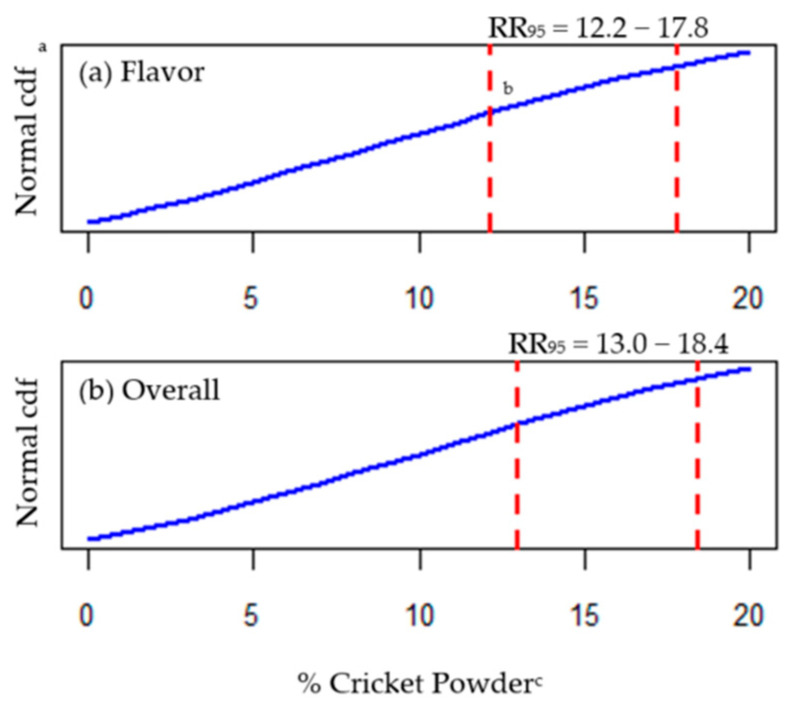
Rejection range_95%_ (RR_95_) for cricket powder in whole-wheat snack crackers. ^^^ RR_95_ was estimated, from a probit regression model, as the range of *x*-values (% cricket powder) bounded by a 95% confidence interval around the RTT_25_ (% cricket powder associated with 25% rejection) for flavor and overall acceptance. ^a^ Normal cumulative distribution functions (cdf) were based on probit regression models, where the left-tailed area under the blue line represents the estimated probability of rejection (acceptability = “no”) as a function of % cricket powder. ^b^ Red dashed vertical lines represent the lower (left) and upper (right) bounds of the RR_95_ for (**a**) flavor and (**b**) overall acceptability, respectively, as % cricket powder. ^c^ Percentage of cricket powder used in place of whole-wheat flour (*w*/*w*) in cracker formulations.

Added value of this methodology is derived from the associated RR which provides the product developer with adaptable formulation guidelines. Based on the inferential nature of a well-fitting probit model, one can calculate a confidence interval around any point-estimate of the RTT with meaningful interpretations [17,18]. We thus defined the RR as the set of stimulus values contained within a confidence interval of interest, surrounding an RTT estimate. Presently, we estimated RR for cricket powder using 95% confidence intervals (RR_95_; Figure 5).

The upper 95% confidence limit for color exceeded 20% cricket powder and, as previously mentioned, RTT_25_ for texture was not determined below 20% cricket powder. Therefore, so as not to interpolate outside the range of data used to fit our models, the RR_95_ is only shown for flavor (12.2%–17.8% cricket powder) and overall acceptability (13.0%–18.4% cricket powder) (Figure 5a,b, respectively). As such, we would predict that 95% of equally sized samples from the same population would reach 25% overall rejection for snack crackers at cricket powder levels between 13% and 18.4% whole-wheat flour substitution, and flavor rejection (at a 25% rejection tolerance) would occur at slightly lower levels (between 12.2% and 17.8%).

Much as with the RTT, it is up to the researcher to choose the appropriate balance of reliability (a confidence level) and precision (width of the confidence interval) in characterizing a pragmatic RR, which may inform profitable decision making. For an ingredient such as cricket powder, which to some extent hinders affective quality, the first reaction may be to simply choose the lower bound of the RR. Instead, the RR should direct strategic adjustments of the product formulation to achieve goals related to cost, quality, health or marketing claims, or nutritional value—all of which decidedly influence consumers’ food choice [34] and ultimately product success.

By narrowing down the stimulus region of interest, more focused testing (e.g., discrimination tests [1,2]) within the RR may reveal opportunities to reduce ingredient costs or maximize nutritional benefits of a product by reaching acceptable upper limits of health-promoting compounds (or cutting back on unhealthy ones) [8,35]. A carefully constructed RR can also be used to set quality control limits for contaminants [3]. For a novel ingredient such as cricket powder, overcoming consumers’ unfamiliarity and expectations of poor sensory quality, which have shown to inhibit trial intent [12], may be achieved through exposure to acceptable products, developed based on consumer-driven data. Furthermore, separate RTT and RR values for different sensory dimensions can guide tailored strategies to improve products that promote sustainability [12,36].

## 4. Conclusions

The present research presented two modified approaches to existing rejection-type threshold methodologies: the M-CRT and M-HRT, as well as two newly proposed thresholds: the RTT and RR. Measures of preference, liking, and acceptance/rejection produced different threshold estimates for cricket powder in whole-wheat snack crackers, as these represent clearly different affective constructs. Terminology used to interpret each threshold should be considered accordingly. As all of the discussed rejection-type threshold methods present value to food research and industry, the authors suggest employing them in the same testing session. Doing so should typically require minimal additional time, sample, cost, and effort on the part of panelists, and provide a more complete view of stimulus effects. Tracking the differential impact of cricket powder on multiple sensory attributes was also valuable to exploring sensory-based limits of a novel ingredient. Other nonlinear approaches, such as Landscape Segmentation Analysis^®^ and internal preference mapping, may be explored in future studies to reveal product attributes which drive acceptance/rejection across multiple segments of consumers.

These results supported existing criticisms of thresholds pertaining to inconsistency in consumers’ perceptual evaluations and lack of congruence between theoretical intent and objective threshold estimates. Rather than relying on statistical significance or some arbitrary boundary between acceptance and rejection, the RTT and RR offered threshold strategies based on practical significance to the investigators or food companies. These concepts are suggested as more realistic approaches to modeling rejection of a food product and offer adaptable methodologies based on a new application of the probit regression model. The RTT can recommend a stimulus level based on an allowable rejection tolerance, and the RR can suggest a stimulus range that will satisfy such a tolerance with a quantifiable level of reliability. These protocols can be used to guide product formulation strategies to meet specific targets in terms of price, quality, nutrition, or functionality and are relevant to various food processing, ingredient, and contaminant effects. Future validation studies should investigate the repeatability of these threshold estimates within a given population.

## Figures and Tables

**Table 1:** Rejection-type threshold concepts.

Threshold Concept	Abbreviation	Reference
Consumer rejection threshold	CRT	Prescott et al. (2005) [3]
Modified consumer rejection threshold	M-CRT	*This study*
Compromised acceptance threshold	CAT	Filjo et al. (2015) [4]
Hedonic rejection threshold	HRT	Filjo et al. (2015; 2018) [4,5]
Modified hedonic rejection threshold	M-HRT	*This study*
Hedonic thresholds methodology	HTM	Filjo et al. (2017) [20]
Hedonic thresholds methodology varying two stimuli	HTM_2s_	Filjo et al. (2020) [6]
Favored acceptance threshold	FAT	Filjo et al. (2020) [6]
Rejection tolerance threshold	RTT	*This study*
Rejection tolerance range	RR	*This study*
